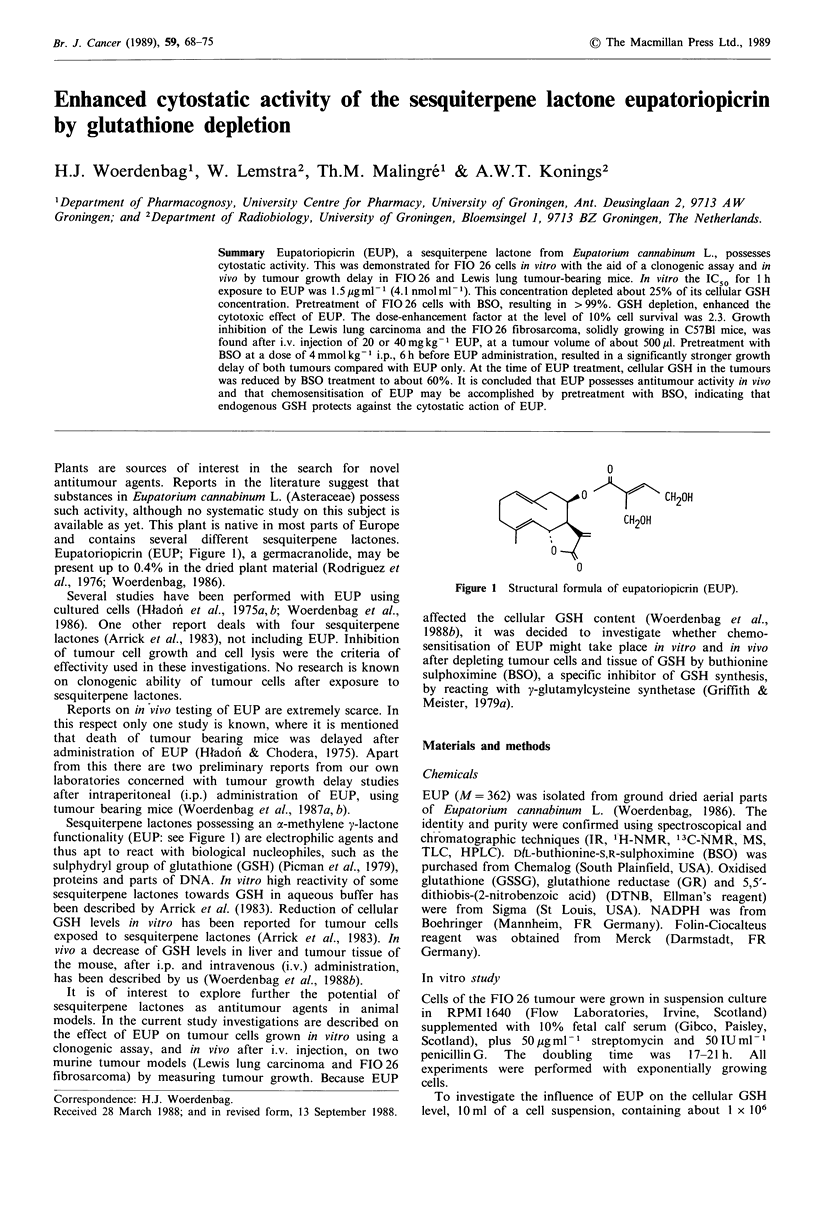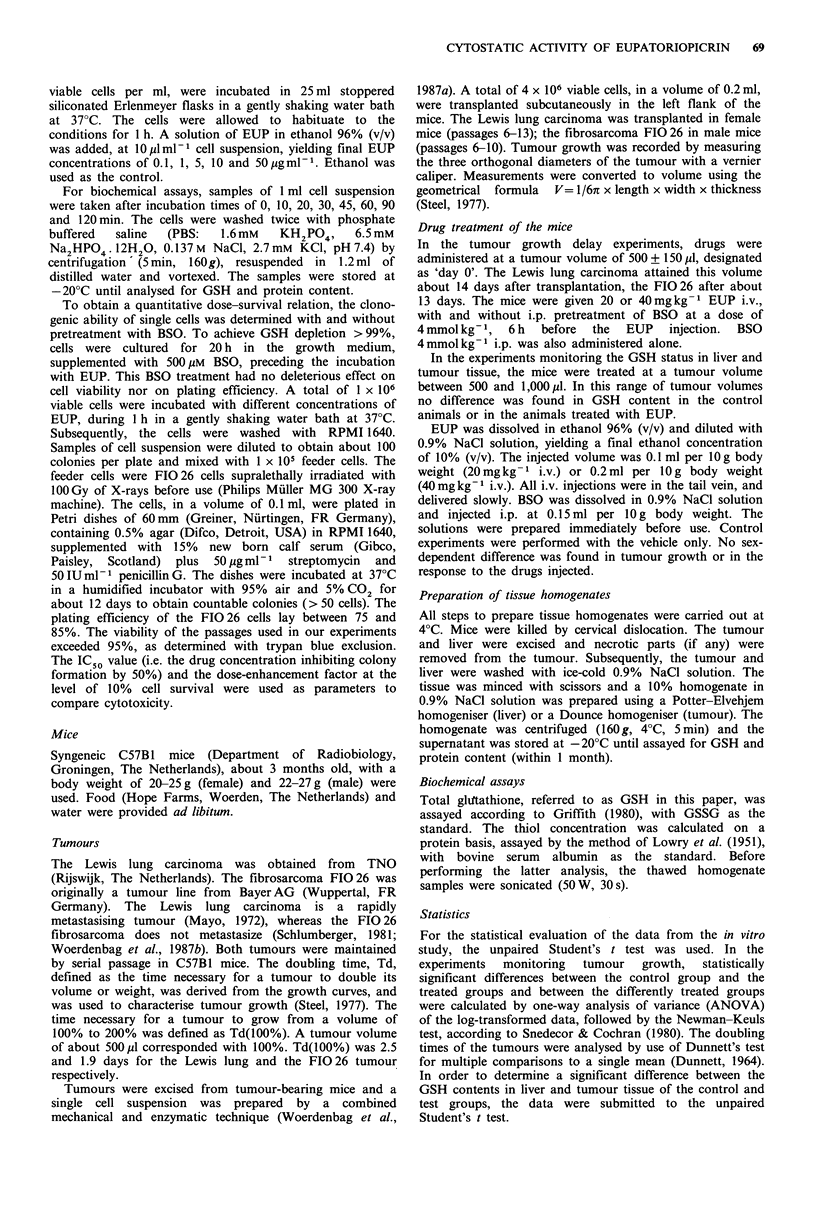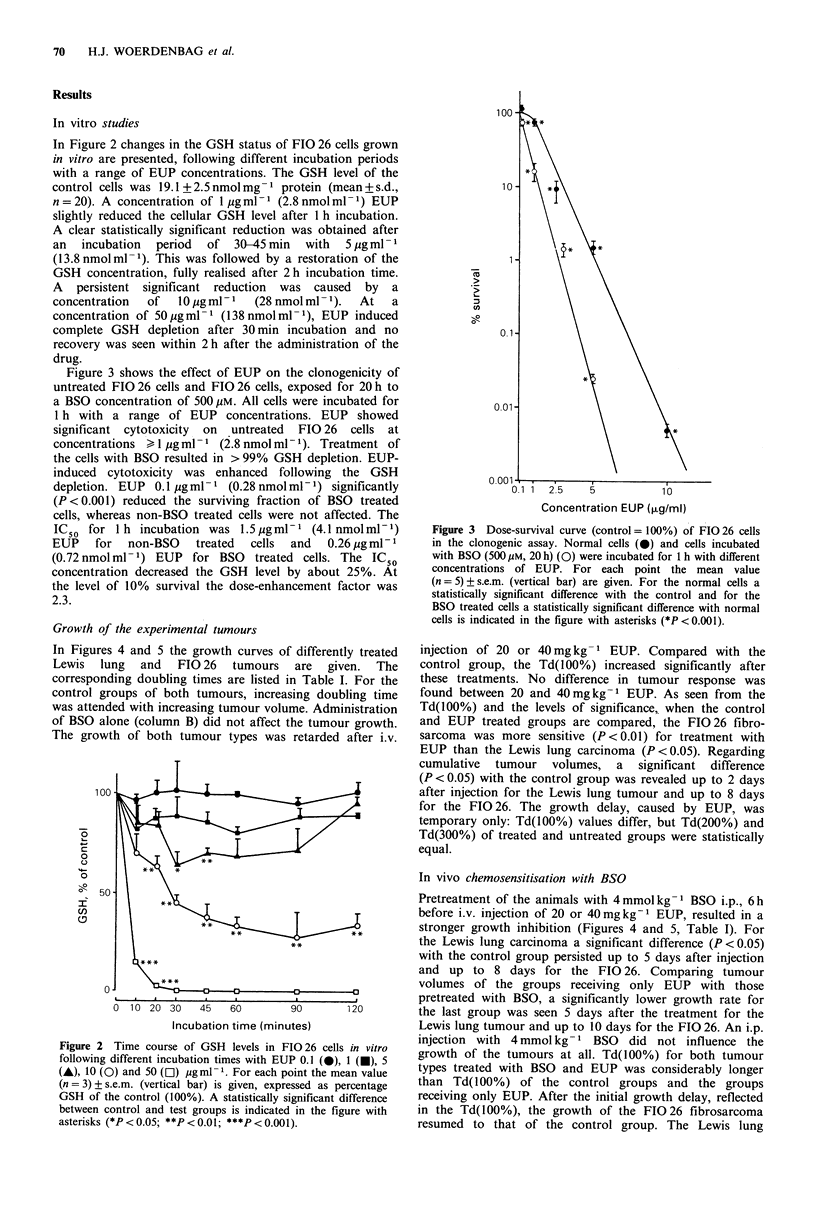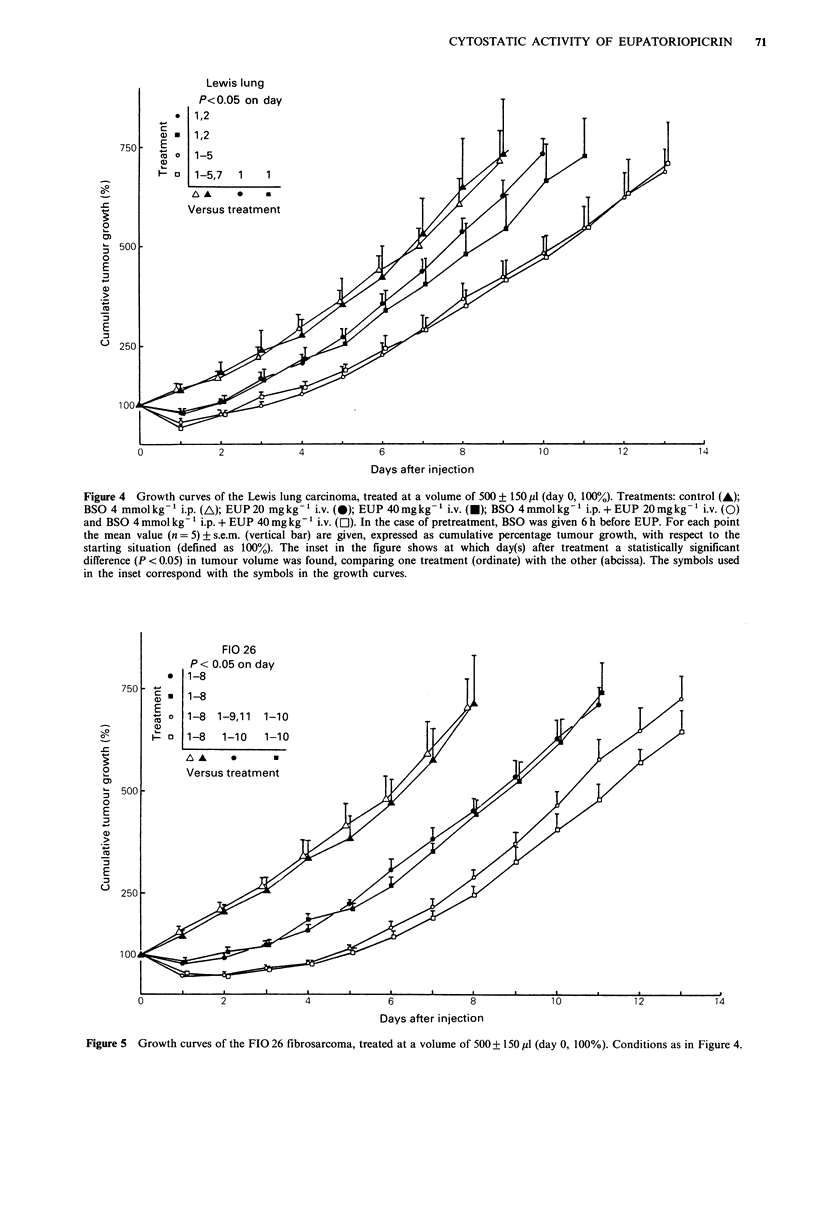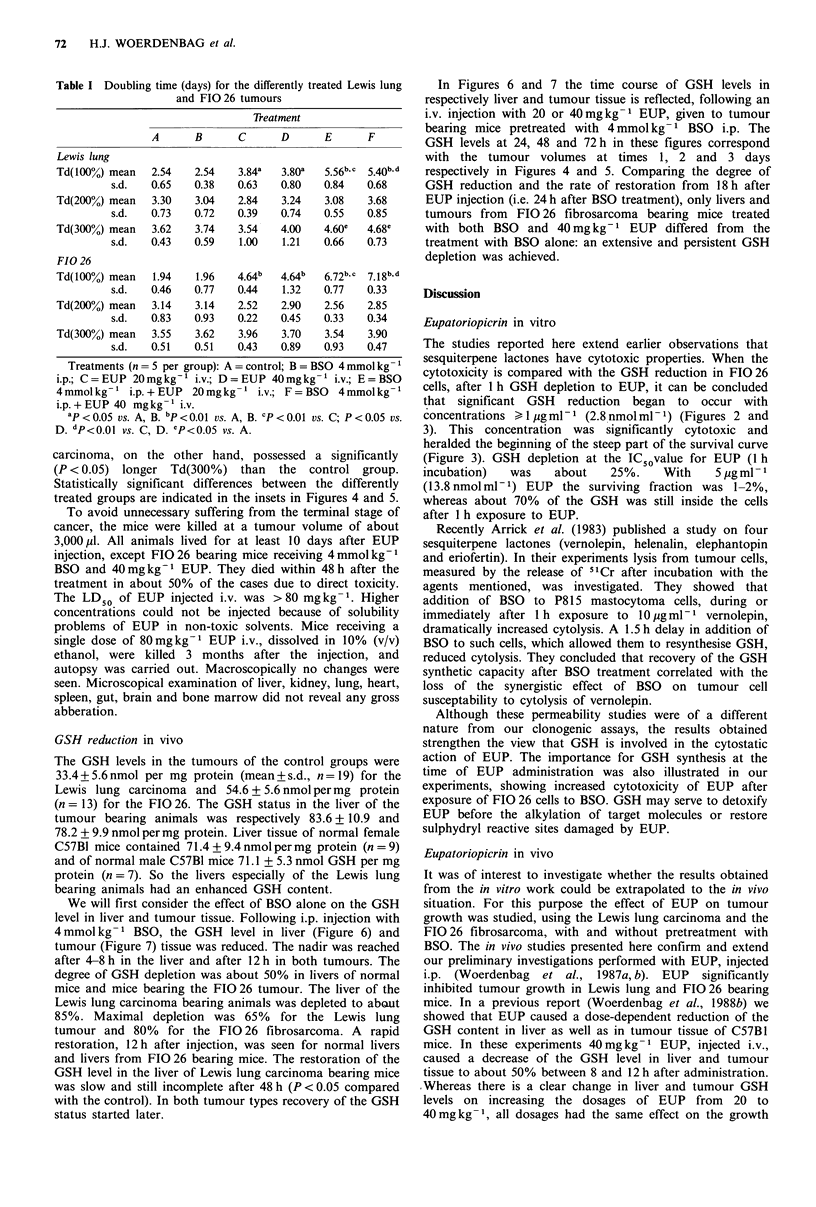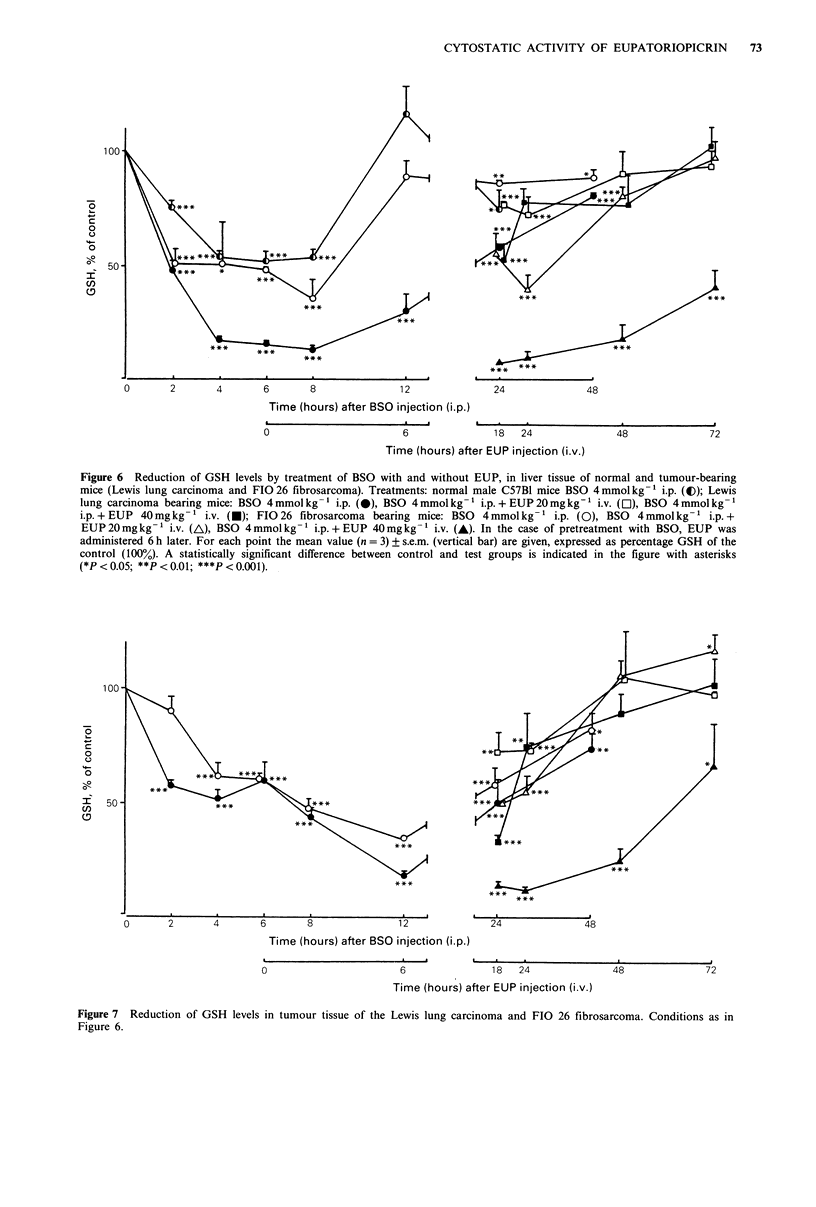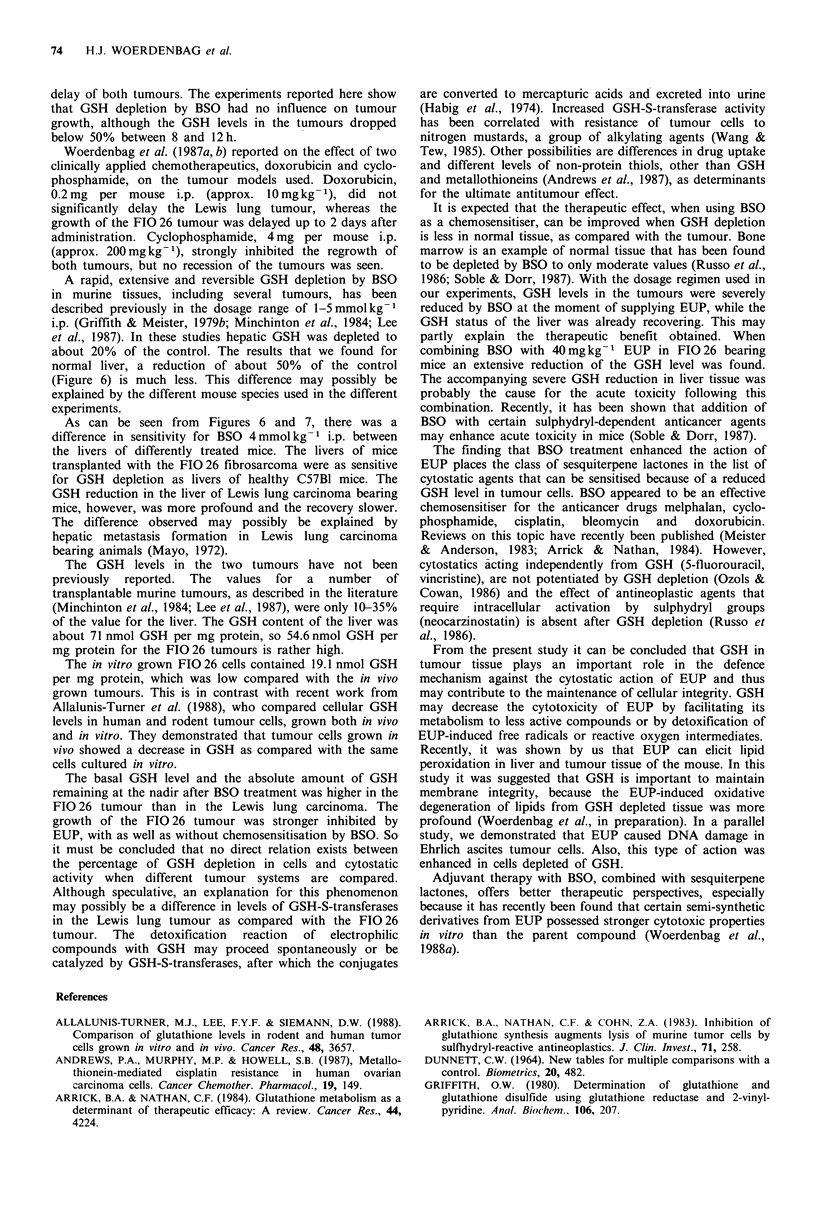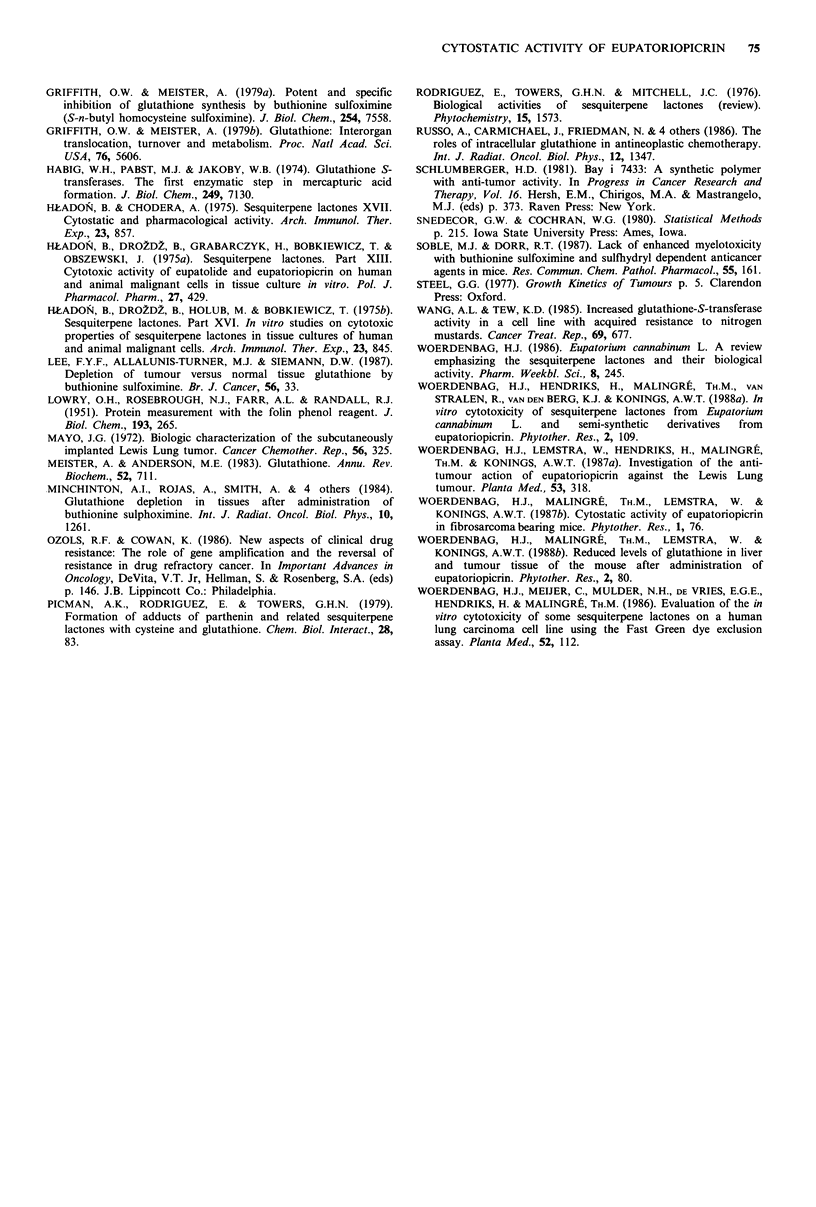# Enhanced cytostatic activity of the sesquiterpene lactone eupatoriopicrin by glutathione depletion.

**DOI:** 10.1038/bjc.1989.13

**Published:** 1989-01

**Authors:** H. J. Woerdenbag, W. Lemstra, T. M. MalingrÃ©, A. W. Konings

**Affiliations:** Department of Pharmacognosy, University Centre for Pharmacy, University of Groningen, The Netherlands.

## Abstract

Eupatoriopicrin (EUP), a sesquiterpene lactone from Eupatorium cannabinum L., possesses cytostatic activity. This was demonstrated for FIO 26 cells in vitro with the aid of a clonogenic assay and in vivo by tumour growth delay in FIO 26 and Lewis lung tumour-bearing mice. In vitro the IC50 for 1 h exposure to EUP was 1.5 microgram ml-1 (4.1 nmol ml-1). This concentration depleted about 25% of its cellular GSH concentration. Pretreatment of FIO 26 cells with BSO, resulting in greater than 99%. GSH depletion, enhanced the cytotoxic effect of EUP. The dose-enhancement factor at the level of 10% cell survival was 2.3. Growth inhibition of the Lewis lung carcinoma and the FIO 26 fibrosarcoma, solidly growing in C57Bl mice, was found after i.v. injection of 20 or 40 mg kg-1 EUP, at a tumour volume of about 500 microliters. Pretreatment with BSO at a dose of 4 mmol kg-1 i.p., 6 h before EUP administration, resulted in a significantly stronger growth delay of both tumours compared with EUP only. At the time of EUP treatment, cellular GSH in the tumours was reduced by BSO treatment to about 60%. It is concluded that EUP possesses antitumour activity in vivo and that chemosensitisation of EUP may be accomplished by pretreatment with BSO, indicating that endogenous GSH protects against the cytostatic action of EUP.


*Br. J. Cancer* (1989), **59**, 68–75 © The Macmillan Press Ltd., 1989

### Enhanced cytostatic activity of the sesquiterpene lactone eupatoriopicrin by glutathione depletion

H.J. Woerdenbag[1], W. Lemstra[2], Th.M. Malingré[1] & A.W.T. Konings[2]

[1]*Department of Pharmacognosy, University Centre for Pharmacy, University of Groningen, Ant. Deusinglaan 2, 9713 AW Groningen; and* [2]*Department of Radiobiology, University of Groningen, Bloemsingel 1, 9713 BZ Groningen, The Netherlands.*

**Summary** Eupatoriopicrin (EUP), a sesquiterpene lactone from *Eupatorium cannabinum* L., possesses cytostatic activity. This was demonstrated for FIO 26 cells *in vitro* with the aid of a clonogenic assay and *in vivo* by tumour growth delay in FIO 26 and Lewis lung tumour-bearing mice. *In vitro* the $IC_{50}$ for 1 h exposure to EUP was 1.5 $\mu g\,ml^{-1}$ (4.1 $nmol\,ml^{-1}$). This concentration depleted about 25% of its cellular GSH concentration. Pretreatment of FIO 26 cells with BSO, resulting in >99%. GSH depletion, enhanced the cytotoxic effect of EUP. The dose-enhancement factor at the level of 10% cell survival was 2.3. Growth inhibition of the Lewis lung carcinoma and the FIO 26 fibrosarcoma, solidly growing in C57Bl mice, was found after i.v. injection of 20 or 40 $mg\,kg^{-1}$ EUP, at a tumour volume of about 500 $\mu l$. Pretreatment with BSO at a dose of 4 $mmol\,kg^{-1}$ i.p., 6 h before EUP administration, resulted in a significantly stronger growth delay of both tumours compared with EUP only. At the time of EUP treatment, cellular GSH in the tumours was reduced by BSO treatment to about 60%. It is concluded that EUP possesses antitumour activity *in vivo* and that chemosensitisation of EUP may be accomplished by pretreatment with BSO, indicating that endogenous GSH protects against the cytostatic action of EUP.

Plants are sources of interest in the search for novel antitumour agents. Reports in the literature suggest that substances in *Eupatorium cannabinum* L. (Asteraceae) possess such activity, although no systematic study on this subject is available as yet. This plant is native in most parts of Europe and contains several different sesquiterpene lactones. Eupatoriopicrin (EUP; Figure 1), a germacranolide, may be present up to 0.4% in the dried plant material (Rodriguez *et al.*, 1976; Woerdenbag, 1986).

Several studies have been performed with EUP using cultured cells (Hładoń *et al.*, 1975*a*, *b*; Woerdenbag *et al.*, 1986). One other report deals with four sesquiterpene lactones (Arrick *et al.*, 1983), not including EUP. Inhibition of tumour cell growth and cell lysis were the criteria of effectivity used in these investigations. No research is known on clonogenic ability of tumour cells after exposure to sesquiterpene lactones.

Reports on *in vivo* testing of EUP are extremely scarce. In this respect only one study is known, where it is mentioned that death of tumour bearing mice was delayed after administration of EUP (Hładoń & Chodera, 1975). Apart from this there are two preliminary reports from our own laboratories concerned with tumour growth delay studies after intraperitoneal (i.p.) administration of EUP, using tumour bearing mice (Woerdenbag *et al.*, 1987*a*, *b*).

Sesquiterpene lactones possessing an α-methylene γ-lactone functionality (EUP: see Figure 1) are electrophilic agents and thus apt to react with biological nucleophiles, such as the sulphydryl group of glutathione (GSH) (Picman *et al.*, 1979), proteins and parts of DNA. *In vitro* high reactivity of some sesquiterpene lactones towards GSH in aqueous buffer has been described by Arrick *et al.* (1983). Reduction of cellular GSH levels *in vitro* has been reported for tumour cells exposed to sesquiterpene lactones (Arrick *et al.*, 1983). *In vivo* a decrease of GSH levels in liver and tumour tissue of the mouse, after i.p. and intravenous (i.v.) administration, has been described by us (Woerdenbag *et al.*, 1988*b*).

It is of interest to explore further the potential of sesquiterpene lactones as antitumour agents in animal models. In the current study investigations are described on the effect of EUP on tumour cells grown *in vitro* using a clonogenic assay, and *in vivo* after i.v. injection, on two murine tumour models (Lewis lung carcinoma and FIO 26 fibrosarcoma) by measuring tumour growth. Because EUP

**Figure 1** Structural formula of eupatoriopicrin (EUP).

affected the cellular GSH content (Woerdenbag *et al.*, 1988*b*), it was decided to investigate whether chemosensitisation of EUP might take place *in vitro* and *in vivo* after depleting tumour cells and tissue of GSH by buthionine sulphoximine (BSO), a specific inhibitor of GSH synthesis, by reacting with γ-glutamylcysteine synthetase (Griffith & Meister, 1979*a*).

### Materials and methods

### *Chemicals*

EUP ($M = 362$) was isolated from ground dried aerial parts of *Eupatorium cannabinum* L. (Woerdenbag, 1986). The identity and purity were confirmed using spectroscopical and chromatographic techniques (IR, $^{1}H$-NMR, $^{13}C$-NMR, MS, TLC, HPLC). D/L-buthionine-S,R-sulphoximine (BSO) was purchased from Chemalog (South Plainfield, USA). Oxidised glutathione (GSSG), glutathione reductase (GR) and 5,5′-dithiobis-(2-nitrobenzoic acid) (DTNB, Ellman's reagent) were from Sigma (St Louis, USA). NADPH was from Boehringer (Mannheim, FR Germany). Folin-Ciocalteus reagent was obtained from Merck (Darmstadt, FR Germany).

### In vitro *study*

Cells of the FIO 26 tumour were grown in suspension culture in RPMI 1640 (Flow Laboratories, Irvine, Scotland) supplemented with 10% fetal calf serum (Gibco, Paisley, Scotland), plus 50 $\mu g\,ml^{-1}$ streptomycin and 50 $IU\,ml^{-1}$ penicillin G. The doubling time was 17–21 h. All experiments were performed with exponentially growing cells.

To investigate the influence of EUP on the cellular GSH level, 10 ml of a cell suspension, containing about $1 \times 10^6$

Correspondence: H.J. Woerdenbag.
Received 28 March 1988; and in revised form, 13 September 1988.

viable cells per ml, were incubated in 25 ml stoppered siliconated Erlenmeyer flasks in a gently shaking water bath at 37°C. The cells were allowed to habituate to the conditions for 1 h. A solution of EUP in ethanol 96% (v/v) was added, at $10\,\mu l\,ml^{-1}$ cell suspension, yielding final EUP concentrations of 0.1, 1, 5, 10 and $50\,\mu g\,ml^{-1}$. Ethanol was used as the control.

For biochemical assays, samples of 1 ml cell suspension were taken after incubation times of 0, 10, 20, 30, 45, 60, 90 and 120 min. The cells were washed twice with phosphate buffered saline (PBS: 1.6 mM $KH_2PO_4$, 6.5 mM $Na_2HPO_4 . 12H_2O$, 0.137 M NaCl, 2.7 mM KCl, pH 7.4) by centrifugation (5 min, 160 *g*), resuspended in 1.2 ml of distilled water and vortexed. The samples were stored at −20°C until analysed for GSH and protein content.

To obtain a quantitative dose–survival relation, the clonogenic ability of single cells was determined with and without pretreatment with BSO. To achieve GSH depletion >99%, cells were cultured for 20 h in the growth medium, supplemented with 500 μM BSO, preceding the incubation with EUP. This BSO treatment had no deleterious effect on cell viability nor on plating efficiency. A total of $1 \times 10^6$ viable cells were incubated with different concentrations of EUP, during 1 h in a gently shaking water bath at 37°C. Subsequently, the cells were washed with RPMI 1640. Samples of cell suspension were diluted to obtain about 100 colonies per plate and mixed with $1 \times 10^5$ feeder cells. The feeder cells were FIO 26 cells supralethally irradiated with 100 Gy of X-rays before use (Philips Müller MG 300 X-ray machine). The cells, in a volume of 0.1 ml, were plated in Petri dishes of 60 mm (Greiner, Nürtingen, FR Germany), containing 0.5% agar (Difco, Detroit, USA) in RPMI 1640, supplemented with 15% new born calf serum (Gibco, Paisley, Scotland) plus $50\,\mu g\,ml^{-1}$ streptomycin and $50\,IU\,ml^{-1}$ penicillin G. The dishes were incubated at 37°C in a humidified incubator with 95% air and 5% $CO_2$ for about 12 days to obtain countable colonies (>50 cells). The plating efficiency of the FIO 26 cells lay between 75 and 85%. The viability of the passages used in our experiments exceeded 95%, as determined with trypan blue exclusion. The $IC_{50}$ value (i.e. the drug concentration inhibiting colony formation by 50%) and the dose-enhancement factor at the level of 10% cell survival were used as parameters to compare cytotoxicity.

*Mice*

Syngeneic C57B1 mice (Department of Radiobiology, Groningen, The Netherlands), about 3 months old, with a body weight of 20–25 g (female) and 22–27 g (male) were used. Food (Hope Farms, Woerden, The Netherlands) and water were provided *ad libitum*.

*Tumours*

The Lewis lung carcinoma was obtained from TNO (Rijswijk, The Netherlands). The fibrosarcoma FIO 26 was originally a tumour line from Bayer AG (Wuppertal, FR Germany). The Lewis lung carcinoma is a rapidly metastasising tumour (Mayo, 1972), whereas the FIO 26 fibrosarcoma does not metastasize (Schlumberger, 1981; Woerdenbag *et al.*, 1987*b*). Both tumours were maintained by serial passage in C57B1 mice. The doubling time, Td, defined as the time necessary for a tumour to double its volume or weight, was derived from the growth curves, and was used to characterise tumour growth (Steel, 1977). The time necessary for a tumour to grow from a volume of 100% to 200% was defined as Td(100%). A tumour volume of about 500 μl corresponded with 100%. Td(100%) was 2.5 and 1.9 days for the Lewis lung and the FIO 26 tumour respectively.

Tumours were excised from tumour-bearing mice and a single cell suspension was prepared by a combined mechanical and enzymatic technique (Woerdenbag *et al.*, 1987*a*). A total of $4 \times 10^6$ viable cells, in a volume of 0.2 ml, were transplanted subcutaneously in the left flank of the mice. The Lewis lung carcinoma was transplanted in female mice (passages 6–13); the fibrosarcoma FIO 26 in male mice (passages 6–10). Tumour growth was recorded by measuring the three orthogonal diameters of the tumour with a vernier caliper. Measurements were converted to volume using the geometrical formula $V = 1/6\pi \times \text{length} \times \text{width} \times \text{thickness}$ (Steel, 1977).

*Drug treatment of the mice*

In the tumour growth delay experiments, drugs were administered at a tumour volume of $500 \pm 150\,\mu l$, designated as 'day 0'. The Lewis lung carcinoma attained this volume about 14 days after transplantation, the FIO 26 after about 13 days. The mice were given 20 or $40\,mg\,kg^{-1}$ EUP i.v., with and without i.p. pretreatment of BSO at a dose of $4\,mmol\,kg^{-1}$, 6 h before the EUP injection. BSO $4\,mmol\,kg^{-1}$ i.p. was also administered alone.

In the experiments monitoring the GSH status in liver and tumour tissue, the mice were treated at a tumour volume between 500 and 1,000 μl. In this range of tumour volumes no difference was found in GSH content in the control animals or in the animals treated with EUP.

EUP was dissolved in ethanol 96% (v/v) and diluted with 0.9% NaCl solution, yielding a final ethanol concentration of 10% (v/v). The injected volume was 0.1 ml per 10 g body weight ($20\,mg\,kg^{-1}$ i.v.) or 0.2 ml per 10 g body weight ($40\,mg\,kg^{-1}$ i.v.). All i.v. injections were in the tail vein, and delivered slowly. BSO was dissolved in 0.9% NaCl solution and injected i.p. at 0.15 ml per 10 g body weight. The solutions were prepared immediately before use. Control experiments were performed with the vehicle only. No sex-dependent difference was found in tumour growth or in the response to the drugs injected.

*Preparation of tissue homogenates*

All steps to prepare tissue homogenates were carried out at 4°C. Mice were killed by cervical dislocation. The tumour and liver were excised and necrotic parts (if any) were removed from the tumour. Subsequently, the tumour and liver were washed with ice-cold 0.9% NaCl solution. The tissue was minced with scissors and a 10% homogenate in 0.9% NaCl solution was prepared using a Potter–Elvehjem homogeniser (liver) or a Dounce homogeniser (tumour). The homogenate was centrifuged (160 *g*, 4°C, 5 min) and the supernatant was stored at −20°C until assayed for GSH and protein content (within 1 month).

*Biochemical assays*

Total glutathione, referred to as GSH in this paper, was assayed according to Griffith (1980), with GSSG as the standard. The thiol concentration was calculated on a protein basis, assayed by the method of Lowry *et al.* (1951), with bovine serum albumin as the standard. Before performing the latter analysis, the thawed homogenate samples were sonicated (50 W, 30 s).

*Statistics*

For the statistical evaluation of the data from the *in vitro* study, the unpaired Student's *t* test was used. In the experiments monitoring tumour growth, statistically significant differences between the control group and the treated groups and between the differently treated groups were calculated by one-way analysis of variance (ANOVA) of the log-transformed data, followed by the Newman–Keuls test, according to Snedecor & Cochran (1980). The doubling times of the tumours were analysed by use of Dunnett's test for multiple comparisons to a single mean (Dunnett, 1964). In order to determine a significant difference between the GSH contents in liver and tumour tissue of the control and test groups, the data were submitted to the unpaired Student's *t* test.

### Results

### In vitro *studies*

In Figure 2 changes in the GSH status of FIO 26 cells grown *in vitro* are presented, following different incubation periods with a range of EUP concentrations. The GSH level of the control cells was $19.1 \pm 2.5$ nmol mg$^{-1}$ protein (mean $\pm$ s.d., $n = 20$). A concentration of 1 $\mu$g ml$^{-1}$ (2.8 nmol ml$^{-1}$) EUP slightly reduced the cellular GSH level after 1 h incubation. A clear statistically significant reduction was obtained after an incubation period of 30–45 min with 5 $\mu$g ml$^{-1}$ (13.8 nmol ml$^{-1}$). This was followed by a restoration of the GSH concentration, fully realised after 2 h incubation time. A persistent significant reduction was caused by a concentration of 10 $\mu$g ml$^{-1}$ (28 nmol ml$^{-1}$). At a concentration of 50 $\mu$g ml$^{-1}$ (138 nmol ml$^{-1}$), EUP induced complete GSH depletion after 30 min incubation and no recovery was seen within 2 h after the administration of the drug.

Figure 3 shows the effect of EUP on the clonogenicity of untreated FIO 26 cells and FIO 26 cells, exposed for 20 h to a BSO concentration of 500 $\mu$M. All cells were incubated for 1 h with a range of EUP concentrations. EUP showed significant cytotoxicity on untreated FIO 26 cells at concentrations $\geqslant 1$ $\mu$g ml$^{-1}$ (2.8 nmol ml$^{-1}$). Treatment of the cells with BSO resulted in $>99\%$ GSH depletion. EUP-induced cytotoxicity was enhanced following the GSH depletion. EUP 0.1 $\mu$g ml$^{-1}$ (0.28 nmol ml$^{-1}$) significantly ($P < 0.001$) reduced the surviving fraction of BSO treated cells, whereas non-BSO treated cells were not affected. The $IC_{50}$ for 1 h incubation was 1.5 $\mu$g ml$^{-1}$ (4.1 nmol ml$^{-1}$) EUP for non-BSO treated cells and 0.26 $\mu$g ml$^{-1}$ (0.72 nmol ml$^{-1}$) EUP for BSO treated cells. The $IC_{50}$ concentration decreased the GSH level by about 25%. At the level of 10% survival the dose-enhancement factor was 2.3.

### *Growth of the experimental tumours*

In Figures 4 and 5 the growth curves of differently treated Lewis lung and FIO 26 tumours are given. The corresponding doubling times are listed in Table I. For the control groups of both tumours, increasing doubling time was attended with increasing tumour volume. Administration of BSO alone (column B) did not affect the tumour growth. The growth of both tumour types was retarded after i.v.

**Figure 2** Time course of GSH levels in FIO 26 cells *in vitro* following different incubation times with EUP 0.1 (●), 1 (■), 5 (▲), 10 (○) and 50 (□) $\mu$g ml$^{-1}$. For each point the mean value ($n = 3$) $\pm$ s.e.m. (vertical bar) is given, expressed as percentage GSH of the control (100%). A statistically significant difference between control and test groups is indicated in the figure with asterisks (*$P < 0.05$; **$P < 0.01$; ***$P < 0.001$).

**Figure 3** Dose-survival curve (control = 100%) of FIO 26 cells in the clonogenic assay. Normal cells (●) and cells incubated with BSO (500 $\mu$M, 20 h) (○) were incubated for 1 h with different concentrations of EUP. For each point the mean value ($n = 5$) $\pm$ s.e.m. (vertical bar) are given. For the normal cells a statistically significant difference with the control and for the BSO treated cells a statistically significant difference with normal cells is indicated in the figure with asterisks (*$P < 0.001$).

injection of 20 or 40 mg kg$^{-1}$ EUP. Compared with the control group, the Td(100%) increased significantly after these treatments. No difference in tumour response was found between 20 and 40 mg kg$^{-1}$ EUP. As seen from the Td(100%) and the levels of significance, when the control and EUP treated groups are compared, the FIO 26 fibrosarcoma was more sensitive ($P < 0.01$) for treatment with EUP than the Lewis lung carcinoma ($P < 0.05$). Regarding cumulative tumour volumes, a significant difference ($P < 0.05$) with the control group was revealed up to 2 days after injection for the Lewis lung tumour and up to 8 days for the FIO 26. The growth delay, caused by EUP, was temporary only: Td(100%) values differ, but Td(200%) and Td(300%) of treated and untreated groups were statistically equal.

### In vivo *chemosensitisation with BSO*

Pretreatment of the animals with 4 mmol kg$^{-1}$ BSO i.p., 6 h before i.v. injection of 20 or 40 mg kg$^{-1}$ EUP, resulted in a stronger growth inhibition (Figures 4 and 5, Table I). For the Lewis lung carcinoma a significant difference ($P < 0.05$) with the control group persisted up to 5 days after injection and up to 8 days for the FIO 26. Comparing tumour volumes of the groups receiving only EUP with those pretreated with BSO, a significantly lower growth rate for the last group was seen 5 days after the treatment for the Lewis lung tumour and up to 10 days for the FIO 26. An i.p. injection with 4 mmol kg$^{-1}$ BSO did not influence the growth of the tumours at all. Td(100%) for both tumour types treated with BSO and EUP was considerably longer than Td(100%) of the control groups and the groups receiving only EUP. After the initial growth delay, reflected in the Td(100%), the growth of the FIO 26 fibrosarcoma resumed to that of the control group. The Lewis lung

**Figure 4** Growth curves of the Lewis lung carcinoma, treated at a volume of 500 ± 150 μl (day 0, 100%). Treatments: control (▲); BSO 4 mmol kg$^{-1}$ i.p. (△); EUP 20 mg kg$^{-1}$ i.v. (●); EUP 40 mg kg$^{-1}$ i.v. (■); BSO 4 mmol kg$^{-1}$ i.p. + EUP 20 mg kg$^{-1}$ i.v. (○) and BSO 4 mmol kg$^{-1}$ i.p. + EUP 40 mg kg$^{-1}$ i.v. (□). In the case of pretreatment, BSO was given 6 h before EUP. For each point the mean value ($n = 5$) ± s.e.m. (vertical bar) are given, expressed as cumulative percentage tumour growth, with respect to the starting situation (defined as 100%). The inset in the figure shows at which day(s) after treatment a statistically significant difference ($P < 0.05$) in tumour volume was found, comparing one treatment (ordinate) with the other (abcissa). The symbols used in the inset correspond with the symbols in the growth curves.

**Figure 5** Growth curves of the FIO 26 fibrosarcoma, treated at a volume of 500 ± 150 μl (day 0, 100%). Conditions as in Figure 4.

**Table I** Doubling time (days) for the differently treated Lewis lung and FIO 26 tumours

| | Treatment | | | | | |
|---|---|---|---|---|---|---|
| | *A* | *B* | *C* | *D* | *E* | *F* |
| *Lewis lung* | | | | | | |
| Td(100%) mean | 2.54 | 2.54 | 3.84[a] | 3.80[a] | 5.56[b,c] | 5.40[b,d] |
| s.d. | 0.65 | 0.38 | 0.63 | 0.80 | 0.84 | 0.68 |
| Td(200%) mean | 3.30 | 3.04 | 2.84 | 3.24 | 3.08 | 3.68 |
| s.d. | 0.73 | 0.72 | 0.39 | 0.74 | 0.55 | 0.85 |
| Td(300%) mean | 3.62 | 3.74 | 3.54 | 4.00 | 4.60[e] | 4.68[e] |
| s.d. | 0.43 | 0.59 | 1.00 | 1.21 | 0.66 | 0.73 |
| *FIO 26* | | | | | | |
| Td(100%) mean | 1.94 | 1.96 | 4.64[b] | 4.64[b] | 6.72[b,c] | 7.18[b,d] |
| s.d. | 0.46 | 0.77 | 0.44 | 1.32 | 0.77 | 0.33 |
| Td(200%) mean | 3.14 | 3.14 | 2.52 | 2.90 | 2.56 | 2.85 |
| s.d. | 0.83 | 0.93 | 0.22 | 0.45 | 0.33 | 0.34 |
| Td(300%) mean | 3.55 | 3.62 | 3.96 | 3.70 | 3.54 | 3.90 |
| s.d. | 0.51 | 0.51 | 0.43 | 0.89 | 0.93 | 0.47 |

Treatments ($n = 5$ per group): A = control; B = BSO 4 mmol kg$^{-1}$ i.p.; C = EUP 20 mg kg$^{-1}$ i.v.; D = EUP 40 mg kg$^{-1}$ i.v.; E = BSO 4 mmol kg$^{-1}$ i.p. + EUP 20 mg kg$^{-1}$ i.v.; F = BSO 4 mmol kg$^{-1}$ i.p. + EUP 40 mg kg$^{-1}$ i.v.

[a]$P < 0.05$ *vs.* A, B. [b]$P < 0.01$ *vs.* A, B. [c]$P < 0.01$ *vs.* C; $P < 0.05$ *vs.* D. [d]$P < 0.01$ *vs.* C, D. [e]$P < 0.05$ *vs.* A.

carcinoma, on the other hand, possessed a significantly ($P < 0.05$) longer Td(300%) than the control group. Statistically significant differences between the differently treated groups are indicated in the insets in Figures 4 and 5.

To avoid unnecessary suffering from the terminal stage of cancer, the mice were killed at a tumour volume of about 3,000 μl. All animals lived for at least 10 days after EUP injection, except FIO 26 bearing mice receiving 4 mmol kg$^{-1}$ BSO and 40 mg kg$^{-1}$ EUP. They died within 48 h after the treatment in about 50% of the cases due to direct toxicity. The $LD_{50}$ of EUP injected i.v. was > 80 mg kg$^{-1}$. Higher concentrations could not be injected because of solubility problems of EUP in non-toxic solvents. Mice receiving a single dose of 80 mg kg$^{-1}$ EUP i.v., dissolved in 10% (v/v) ethanol, were killed 3 months after the injection, and autopsy was carried out. Macroscopically no changes were seen. Microscopical examination of liver, kidney, lung, heart, spleen, gut, brain and bone marrow did not reveal any gross abberation.

*GSH reduction* in vivo

The GSH levels in the tumours of the control groups were 33.4 ± 5.6 nmol per mg protein (mean ± s.d., $n = 19$) for the Lewis lung carcinoma and 54.6 ± 5.6 nmol per mg protein ($n = 13$) for the FIO 26. The GSH status in the liver of the tumour bearing animals was respectively 83.6 ± 10.9 and 78.2 ± 9.9 nmol per mg protein. Liver tissue of normal female C57Bl mice contained 71.4 ± 9.4 nmol per mg protein ($n = 9$) and of normal male C57Bl mice 71.1 ± 5.3 nmol GSH per mg protein ($n = 7$). So the livers especially of the Lewis lung bearing animals had an enhanced GSH content.

We will first consider the effect of BSO alone on the GSH level in liver and tumour tissue. Following i.p. injection with 4 mmol kg$^{-1}$ BSO, the GSH level in liver (Figure 6) and tumour (Figure 7) tissue was reduced. The nadir was reached after 4–8 h in the liver and after 12 h in both tumours. The degree of GSH depletion was about 50% in livers of normal mice and mice bearing the FIO 26 tumour. The liver of the Lewis lung carcinoma bearing animals was depleted to about 85%. Maximal depletion was 65% for the Lewis lung tumour and 80% for the FIO 26 fibrosarcoma. A rapid restoration, 12 h after injection, was seen for normal livers and livers from FIO 26 bearing mice. The restoration of the GSH level in the liver of Lewis lung carcinoma bearing mice was slow and still incomplete after 48 h ($P < 0.05$ compared with the control). In both tumour types recovery of the GSH status started later.

In Figures 6 and 7 the time course of GSH levels in respectively liver and tumour tissue is reflected, following an i.v. injection with 20 or 40 mg kg$^{-1}$ EUP, given to tumour bearing mice pretreated with 4 mmol kg$^{-1}$ BSO i.p. The GSH levels at 24, 48 and 72 h in these figures correspond with the tumour volumes at times 1, 2 and 3 days respectively in Figures 4 and 5. Comparing the degree of GSH reduction and the rate of restoration from 18 h after EUP injection (i.e. 24 h after BSO treatment), only livers and tumours from FIO 26 fibrosarcoma bearing mice treated with both BSO and 40 mg kg$^{-1}$ EUP differed from the treatment with BSO alone: an extensive and persistent GSH depletion was achieved.

### Discussion

*Eupatoriopicrin* in vitro

The studies reported here extend earlier observations that sesquiterpene lactones have cytotoxic properties. When the cytotoxicity is compared with the GSH reduction in FIO 26 cells, after 1 h GSH depletion to EUP, it can be concluded that significant GSH reduction began to occur with concentrations ⩾ 1 μg ml$^{-1}$ (2.8 nmol ml$^{-1}$) (Figures 2 and 3). This concentration was significantly cytotoxic and heralded the beginning of the steep part of the survival curve (Figure 3). GSH depletion at the $IC_{50}$value for EUP (1 h incubation) was about 25%. With 5 μg ml$^{-1}$ (13.8 nmol ml$^{-1}$) EUP the surviving fraction was 1–2%, whereas about 70% of the GSH was still inside the cells after 1 h exposure to EUP.

Recently Arrick *et al.* (1983) published a study on four sesquiterpene lactones (vernolepin, helenalin, elephantopin and eriofertin). In their experiments lysis from tumour cells, measured by the release of $^{51}Cr$ after incubation with the agents mentioned, was investigated. They showed that addition of BSO to P815 mastocytoma cells, during or immediately after 1 h exposure to 10 μg ml$^{-1}$ vernolepin, dramatically increased cytolysis. A 1.5 h delay in addition of BSO to such cells, which allowed them to resynthesise GSH, reduced cytolysis. They concluded that recovery of the GSH synthetic capacity after BSO treatment correlated with the loss of the synergistic effect of BSO on tumour cell susceptability to cytolysis of vernolepin.

Although these permeability studies were of a different nature from our clonogenic assays, the results obtained strengthen the view that GSH is involved in the cytostatic action of EUP. The importance for GSH synthesis at the time of EUP administration was also illustrated in our experiments, showing increased cytotoxicity of EUP after exposure of FIO 26 cells to BSO. GSH may serve to detoxify EUP before the alkylation of target molecules or restore sulphydryl reactive sites damaged by EUP.

*Eupatoriopicrin* in vivo

It was of interest to investigate whether the results obtained from the *in vitro* work could be extrapolated to the *in vivo* situation. For this purpose the effect of EUP on tumour growth was studied, using the Lewis lung carcinoma and the FIO 26 fibrosarcoma, with and without pretreatment with BSO. The *in vivo* studies presented here confirm and extend our preliminary investigations performed with EUP, injected i.p. (Woerdenbag *et al.*, 1987*a*, *b*). EUP significantly inhibited tumour growth in Lewis lung and FIO 26 bearing mice. In a previous report (Woerdenbag *et al.*, 1988*b*) we showed that EUP caused a dose-dependent reduction of the GSH content in liver as well as in tumour tissue of C57Bl mice. In these experiments 40 mg kg$^{-1}$ EUP, injected i.v., caused a decrease of the GSH level in liver and tumour tissue to about 50% between 8 and 12 h after administration. Whereas there is a clear change in liver and tumour GSH levels on increasing the dosages of EUP from 20 to 40 mg kg$^{-1}$, all dosages had the same effect on the growth

**Figure 6** Reduction of GSH levels by treatment of BSO with and without EUP, in liver tissue of normal and tumour-bearing mice (Lewis lung carcinoma and FIO 26 fibrosarcoma). Treatments: normal male C57Bl mice BSO 4 mmol kg$^{-1}$ i.p. (◐); Lewis lung carcinoma bearing mice: BSO 4 mmol kg$^{-1}$ i.p. (●), BSO 4 mmol kg$^{-1}$ i.p. + EUP 20 mg kg$^{-1}$ i.v. (□), BSO 4 mmol kg$^{-1}$ i.p. + EUP 40 mg kg$^{-1}$ i.v. (■); FIO 26 fibrosarcoma bearing mice: BSO 4 mmol kg$^{-1}$ i.p. (○), BSO 4 mmol kg$^{-1}$ i.p. + EUP 20 mg kg$^{-1}$ i.v. (△), BSO 4 mmol kg$^{-1}$ i.p. + EUP 40 mg kg$^{-1}$ i.v. (▲). In the case of pretreatment with BSO, EUP was administered 6 h later. For each point the mean value ($n = 3$) ± s.e.m. (vertical bar) are given, expressed as percentage GSH of the control (100%). A statistically significant difference between control and test groups is indicated in the figure with asterisks (*$P < 0.05$; **$P < 0.01$; ***$P < 0.001$).

**Figure 7** Reduction of GSH levels in tumour tissue of the Lewis lung carcinoma and FIO 26 fibrosarcoma. Conditions as in Figure 6.

delay of both tumours. The experiments reported here show that GSH depletion by BSO had no influence on tumour growth, although the GSH levels in the tumours dropped below 50% between 8 and 12 h.

Woerdenbag *et al.* (1987*a*, *b*) reported on the effect of two clinically applied chemotherapeutics, doxorubicin and cyclophosphamide, on the tumour models used. Doxorubicin, 0.2 mg per mouse i.p. (approx. 10 mg $kg^{-1}$), did not significantly delay the Lewis lung tumour, whereas the growth of the FIO 26 tumour was delayed up to 2 days after administration. Cyclophosphamide, 4 mg per mouse i.p. (approx. 200 mg $kg^{-1}$), strongly inhibited the regrowth of both tumours, but no recession of the tumours was seen.

A rapid, extensive and reversible GSH depletion by BSO in murine tissues, including several tumours, has been described previously in the dosage range of 1–5 mmol $kg^{-1}$ i.p. (Griffith & Meister, 1979*b*; Minchinton *et al.*, 1984; Lee *et al.*, 1987). In these studies hepatic GSH was depleted to about 20% of the control. The results that we found for normal liver, a reduction of about 50% of the control (Figure 6) is much less. This difference may possibly be explained by the different mouse species used in the different experiments.

As can be seen from Figures 6 and 7, there was a difference in sensitivity for BSO 4 mmol $kg^{-1}$ i.p. between the livers of differently treated mice. The livers of mice transplanted with the FIO 26 fibrosarcoma were as sensitive for GSH depletion as livers of healthy C57Bl mice. The GSH reduction in the liver of Lewis lung carcinoma bearing mice, however, was more profound and the recovery slower. The difference observed may possibly be explained by hepatic metastasis formation in Lewis lung carcinoma bearing animals (Mayo, 1972).

The GSH levels in the two tumours have not been previously reported. The values for a number of transplantable murine tumours, as described in the literature (Minchinton *et al.*, 1984; Lee *et al.*, 1987), were only 10–35% of the value for the liver. The GSH content of the liver was about 71 nmol GSH per mg protein, so 54.6 nmol GSH per mg protein for the FIO 26 tumours is rather high.

The *in vitro* grown FIO 26 cells contained 19.1 nmol GSH per mg protein, which was low compared with the *in vivo* grown tumours. This is in contrast with recent work from Allalunis-Turner *et al.* (1988), who compared cellular GSH levels in human and rodent tumour cells, grown both *in vivo* and *in vitro*. They demonstrated that tumour cells grown *in vivo* showed a decrease in GSH as compared with the same cells cultured *in vitro*.

The basal GSH level and the absolute amount of GSH remaining at the nadir after BSO treatment was higher in the FIO 26 tumour than in the Lewis lung carcinoma. The growth of the FIO 26 tumour was stronger inhibited by EUP, with as well as without chemosensitisation by BSO. So it must be concluded that no direct relation exists between the percentage of GSH depletion in cells and cytostatic activity when different tumour systems are compared. Although speculative, an explanation for this phenomenon may possibly be a difference in levels of GSH-S-transferases in the Lewis lung tumour as compared with the FIO 26 tumour. The detoxification reaction of electrophilic compounds with GSH may proceed spontaneously or be catalyzed by GSH-S-transferases, after which the conjugates are converted to mercapturic acids and excreted into urine (Habig *et al.*, 1974). Increased GSH-S-transferase activity has been correlated with resistance of tumour cells to nitrogen mustards, a group of alkylating agents (Wang & Tew, 1985). Other possibilities are differences in drug uptake and different levels of non-protein thiols, other than GSH and metallothioneins (Andrews *et al.*, 1987), as determinants for the ultimate antitumour effect.

It is expected that the therapeutic effect, when using BSO as a chemosensitiser, can be improved when GSH depletion is less in normal tissue, as compared with the tumour. Bone marrow is an example of normal tissue that has been found to be depleted by BSO to only moderate values (Russo *et al.*, 1986; Soble & Dorr, 1987). With the dosage regimen used in our experiments, GSH levels in the tumours were severely reduced by BSO at the moment of supplying EUP, while the GSH status of the liver was already recovering. This may partly explain the therapeutic benefit obtained. When combining BSO with 40 mg $kg^{-1}$ EUP in FIO 26 bearing mice an extensive reduction of the GSH level was found. The accompanying severe GSH reduction in liver tissue was probably the cause for the acute toxicity following this combination. Recently, it has been shown that addition of BSO with certain sulphydryl-dependent anticancer agents may enhance acute toxicity in mice (Soble & Dorr, 1987).

The finding that BSO treatment enhanced the action of EUP places the class of sesquiterpene lactones in the list of cytostatic agents that can be sensitised because of a reduced GSH level in tumour cells. BSO appeared to be an effective chemosensitiser for the anticancer drugs melphalan, cyclophosphamide, cisplatin, bleomycin and doxorubicin. Reviews on this topic have recently been published (Meister & Anderson, 1983; Arrick & Nathan, 1984). However, cytostatics acting independently from GSH (5-fluorouracil, vincristine), are not potentiated by GSH depletion (Ozols & Cowan, 1986) and the effect of antineoplastic agents that require intracellular activation by sulphydryl groups (neocarzinostatin) is absent after GSH depletion (Russo *et al.*, 1986).

From the present study it can be concluded that GSH in tumour tissue plays an important role in the defence mechanism against the cytostatic action of EUP and thus may contribute to the maintenance of cellular integrity. GSH may decrease the cytotoxicity of EUP by facilitating its metabolism to less active compounds or by detoxification of EUP-induced free radicals or reactive oxygen intermediates. Recently, it was shown by us that EUP can elicit lipid peroxidation in liver and tumour tissue of the mouse. In this study it was suggested that GSH is important to maintain membrane integrity, because the EUP-induced oxidative degeneration of lipids from GSH depleted tissue was more profound (Woerdenbag *et al.*, in preparation). In a parallel study, we demonstrated that EUP caused DNA damage in Ehrlich ascites tumour cells. Also, this type of action was enhanced in cells depleted of GSH.

Adjuvant therapy with BSO, combined with sesquiterpene lactones, offers better therapeutic perspectives, especially because it has recently been found that certain semi-synthetic derivatives from EUP possessed stronger cytotoxic properties *in vitro* than the parent compound (Woerdenbag *et al.*, 1988*a*).

### References

ALLALUNIS-TURNER, M.J., LEE, F.Y.F. & SIEMANN, D.W. (1988). Comparison of glutathione levels in rodent and human tumor cells grown *in vitro* and *in vivo*. *Cancer Res.*, **48**, 3657.

ANDREWS, P.A., MURPHY, M.P. & HOWELL, S.B. (1987), Metallothionein-mediated cisplatin resistance in human ovarian carcinoma cells. *Cancer Chemother. Pharmacol.*, **19**, 149.

ARRICK, B.A. & NATHAN, C.F. (1984). Glutathione metabolism as a determinant of therapeutic efficacy: A review. *Cancer Res.*, **44**, 4224.

ARRICK, B.A., NATHAN, C.F. & COHN, Z.A. (1983). Inhibition of glutathione synthesis augments lysis of murine tumor cells by sulfhydryl-reactive antineoplastics. *J. Clin. Invest.*, **71**, 258.

DUNNETT, C.W. (1964). New tables for multiple comparisons with a control. *Biometrics*, **20**, 482.

GRIFFITH, O.W. (1980). Determination of glutathione and glutathione disulfide using glutathione reductase and 2-vinylpyridine. *Anal. Biochem.*, **106**, 207.

GRIFFITH, O.W. & MEISTER, A. (1979*a*). Potent and specific inhibition of glutathione synthesis by buthionine sulfoximine (*S-n*-butyl homocysteine sulfoximine). *J. Biol. Chem.*, **254**, 7558.

GRIFFITH, O.W. & MEISTER, A. (1979*b*). Glutathione: Interorgan translocation, turnover and metabolism. *Proc. Natl Acad. Sci. USA*, **76**, 5606.

HABIG, W.H., PABST, M.J. & JAKOBY, W.B. (1974). Glutathione *S*-transferases. The first enzymatic step in mercapturic acid formation. *J. Biol. Chem.*, **249**, 7130.

HŁADOŃ, B. & CHODERA, A. (1975). Sesquiterpene lactones XVII. Cytostatic and pharmacological activity. *Arch. Immunol. Ther. Exp.*, **23**, 857.

HŁADOŃ, B., DROŽDŽ, B., GRABARCZYK, H., BOBKIEWICZ, T. & OBSZEWSKI, J. (1975*a*). Sesquiterpene lactones. Part XIII. Cytotoxic activity of eupatolide and eupatoriopicrin on human and animal malignant cells in tissue culture *in vitro*. *Pol. J. Pharmacol. Pharm.*, **27**, 429.

HŁADOŃ, B., DROŽDŽ, B., HOLUB, M. & BOBKIEWICZ, T. (1975*b*). Sesquiterpene lactones. Part XVI. *In vitro* studies on cytotoxic properties of sesquiterpene lactones in tissue cultures of human and animal malignant cells. *Arch. Immunol. Ther. Exp.*, **23**, 845.

LEE, F.Y.F., ALLALUNIS-TURNER, M.J. & SIEMANN, D.W. (1987). Depletion of tumour versus normal tissue glutathione by buthionine sulfoximine. *Br. J. Cancer*, **56**, 33.

LOWRY, O.H., ROSEBROUGH, N.J., FARR, A.L. & RANDALL, R.J. (1951). Protein measurement with the folin phenol reagent. *J. Biol. Chem.*, **193**, 265.

MAYO, J.G. (1972). Biologic characterization of the subcutaneously implanted Lewis Lung tumor. *Cancer Chemother. Rep.*, **56**, 325.

MEISTER, A. & ANDERSON, M.E. (1983). Glutathione. *Annu. Rev. Biochem.*, **52**, 711.

MINCHINTON, A.I., ROJAS, A., SMITH, A. & 4 others (1984). Glutathione depletion in tissues after administration of buthionine sulphoximine. *Int. J. Radiat. Oncol. Biol. Phys.*, **10**, 1261.

OZOLS, R.F. & COWAN, K. (1986). New aspects of clinical drug resistance: The role of gene amplification and the reversal of resistance in drug refractory cancer. In *Important Advances in Oncology*, DeVita, V.T. Jr, Hellman, S. & Rosenberg, S.A. (eds) p. 146. J.B. Lippincott Co.: Philadelphia.

PICMAN, A.K., RODRIGUEZ, E. & TOWERS, G.H.N. (1979). Formation of adducts of parthenin and related sesquiterpene lactones with cysteine and glutathione. *Chem. Biol. Interact.*, **28**, 83.

RODRIGUEZ, E., TOWERS, G.H.N. & MITCHELL, J.C. (1976). Biological activities of sesquiterpene lactones (review). *Phytochemistry*, **15**, 1573.

RUSSO, A., CARMICHAEL, J., FRIEDMAN, N. & 4 others (1986). The roles of intracellular glutathione in antineoplastic chemotherapy. *Int. J. Radiat. Oncol. Biol. Phys.*, **12**, 1347.

SCHLUMBERGER, H.D. (1981). Bay i 7433: A synthetic polymer with anti-tumor activity. In *Progress in Cancer Research and Therapy, Vol. 16*. Hersh, E.M., Chirigos, M.A. & Mastrangelo, M.J. (eds) p. 373. Raven Press: New York.

SNEDECOR, G.W. & COCHRAN, W.G. (1980). *Statistical Methods* p. 215. Iowa State University Press: Ames, Iowa.

SOBLE, M.J. & DORR, R.T. (1987). Lack of enhanced myelotoxicity with buthionine sulfoximine and sulfhydryl dependent anticancer agents in mice. *Res. Commun. Chem. Pathol. Pharmacol.*, **55**, 161.

STEEL, G.G. (1977). *Growth Kinetics of Tumours* p. 5. Clarendon Press: Oxford.

WANG, A.L. & TEW, K.D. (1985). Increased glutathione-*S*-transferase activity in a cell line with acquired resistance to nitrogen mustards. *Cancer Treat. Rep.*, **69**, 677.

WOERDENBAG, H.J. (1986). *Eupatorium cannabinum* L. A review emphasizing the sesquiterpene lactones and their biological activity. *Pharm. Weekbl. Sci.*, **8**, 245.

WOERDENBAG, H.J., HENDRIKS, H., MALINGRÉ, TH.M., VAN STRALEN, R., VAN DEN BERG, K.J. & KONINGS, A.W.T. (1988*a*). *In vitro* cytotoxicity of sesquiterpene lactones from *Eupatorium cannabinum* L. and semi-synthetic derivatives from eupatoriopicrin. *Phytother. Res.*, **2**, 109.

WOERDENBAG, H.J., LEMSTRA, W., HENDRIKS, H., MALINGRÉ, TH.M. & KONINGS, A.W.T. (1987*a*). Investigation of the anti-tumour action of eupatoriopicrin against the Lewis Lung tumour. *Planta Med.*, **53**, 318.

WOERDENBAG, H.J., MALINGRÉ, TH.M., LEMSTRA, W. & KONINGS, A.W.T. (1987*b*). Cytostatic activity of eupatoriopicrin in fibrosarcoma bearing mice. *Phytother. Res.*, **1**, 76.

WOERDENBAG, H.J., MALINGRÉ, TH.M., LEMSTRA, W. & KONINGS, A.W.T. (1988*b*). Reduced levels of glutathione in liver and tumour tissue of the mouse after administration of eupatoriopicrin. *Phytother. Res.*, **2**, 80.

WOERDENBAG, H.J., MEIJER, C., MULDER, N.H., DE VRIES, E.G.E., HENDRIKS, H. & MALINGRÉ, TH.M. (1986). Evaluation of the *in vitro* cytotoxicity of some sesquiterpene lactones on a human lung carcinoma cell line using the Fast Green dye exclusion assay. *Planta Med.*, **52**, 112.